# Swine Gastrointestinal Microbiota and the Effects of Dietary Amino Acids on Its Composition and Metabolism

**DOI:** 10.3390/ijms25021237

**Published:** 2024-01-19

**Authors:** Shengfa F. Liao, Feng Ji, Peixin Fan, Kristin Denryter

**Affiliations:** 1 Department of Animal and Dairy Sciences, Mississippi State University, Starkville, MS 39762, USA; pf324@msstate.edu (P.F.);; 2 Institute of Animal Husbandry and Veterinary Medicine, Beijing Academy of Agriculture and Forestry Sciences, Beijing 100097, China; jifengji@sina.com

**Keywords:** gastrointestinal tract, microbiota, metabolism, amino acid, swine

## Abstract

Many researchers consider gut microbiota (trillions of microorganisms) an endogenous organ of its animal host, which confers a vast genetic diversity in providing the host with essential biological functions. Particularly, the gut microbiota regulates not only gut tissue structure but also gut health and gut functionality. This paper first summarized those common bacterial species (dominated by the *Firmicutes*, *Bacteroidota*, and *Proteobacteria* phyla) in swine gut and then briefly discussed their roles in swine nutrition and health, which include roles in nutrient metabolism, pathogen exclusion, and immunity modulation. Secondly, the current knowledge on how dietary nutrients and feed additives affect the gut bacterial composition and nutrient metabolism in pigs was discussed. Finally, how dietary amino acids affect the relative abundances and metabolism of bacteria in the swine gut was reviewed. Tryptophan supplementation promotes the growth of beneficial bacteria and suppresses pathogens, while arginine metabolism affects nitrogen recycling, impacting gut immune response and health. Glutamate and glutamine supplementations elevate the levels of beneficial bacteria and mitigate pathogenic ones. It was concluded that nutritional strategies to manipulate gut microbial ecosystems are useful measures to optimize gut health and gut functions. For example, providing pigs with nutrients that promote the growth of *Lactobacillus* and *Bifidobacterium* can lead to better gut health and growth performance, especially when dietary protein is limited. Further research to establish the mechanistic cause-and-effect relationships between amino acids and the dynamics of gut microbiota will allow swine producers to reap the greatest return on their feed investment.

## 1. Introduction

Animals encounter billions of species of microorganisms during their lifetimes, and many particular species can find an appropriate niche in an animal body to establish a long-term close association between them. This type of living association between microbial species and their host is called symbiosis, which can actually present in a commensalistic, mutualistic, or parasitic relationship [1]. Dysbiosis, a disruption to the microbiota homeostasis, on the other hand, can play a detrimental role in chronic diseases such as inflammatory bowel diseases. Although they have evolved to their living environment (especially the feeds), mammalian pigs do rely on a large and diverse community of microorganisms (called microbiota), especially those in the gastrointestinal tract (GIT, or gut), to sustain their lives.

Gut microbiota is primarily comprised of bacteria, but fungi (such as yeasts) and protozoa also comprise significant parts of the community, with ratios depending on the types of animals. For typical mammals, the gut microbiota contains orders of magnitude more cells than the body cells of the host. The microbiome, the collective genome of microbiota, is more than 130 times the size of the host genome [2,3]. This large microbiome confers a vast genetic diversity that provides animals with access to additional biological activities that are otherwise unattainable [4]. A plethora of studies have shown that gut microbiota can provide multifaceted functionality to pigs, especially in the aspects of gut structural integrity, mucosal barrier, immunomodulation, and defense against pathogens, as well as the metabolism of nutrients and xenobiotics.

Additionally, gut microorganisms also play roles in recycling bile salts and hence in fat utilization [5,6]; they also synthesize several B vitamins as well as vitamin K [7,8,9], and assist with host immunity, including by preventing pathogenic microbial species from becoming established [10,11,12]. Thus, gut microbiota is extremely important to swine health, well-being, feed utilization, and production performance [13,14]. This review first summarizes the common microbial species in the swine gut and briefly discusses their roles in swine nutrition and health. The second part of this paper is to review some current knowledge about how dietary nutritional components, especially amino acids (**AA**), affect the gut microbial composition and metabolism in pigs.

*Data acquisition and study inclusion criteria*: Besides the NCBI PubMed and Google Scholar, the literature used for this review was mainly collected through the Advanced Search of the “Web of Science Core Collection” database. The key terms for the topic query include (gastrointestinal tract, gut or intestinal) AND (porcine, sow, swine or pig) AND (microbiome or microbiota) AND (amino acid). No publication-year limit was imposed, and the search was conducted in July 2023 for the last time. The search resulted in 362 scientific articles, of which 73 most-related articles, relevant to “how dietary AA affect the gut microbial composition and metabolism in pigs,” were selected as key articles for the review in this paper.

## 2. Normal Gut Microbiota Composition of Swine

Collaborating in the gut structure maturation and function maintenance are trillions of microbes that reside in it [15], although the taxonomical composition is not static but presents spatial variation along the gut sections and temporal variation at different growth stages of pigs. Historically, the composition of gut microbiota was commonly evaluated based on the traditional culturing techniques (selective plating and biochemical and morphological assays), but the majority (40~90%) of the microbial species in the gut are still unknown or unculturable [8,16]. Recently, the microbiota was commonly quantified using numerous molecular techniques, such as the in-depth next generation sequencing of 16S ribosomal RNA genes or the whole  genome  shotgun  sequencing [16]. That being said, a publicly available repository of the culturable strains of commensal bacteria, called ‘Pig intestinal bacterial collection’ (PiBAC; www.dsmz.de/pibac (accessed on 1 November 2023)), is available, which opened a new avenue for functional studies of swine gut microbiota [17].

A “normal” microbiota composition of pig gut (Supplemental Table S1) can be described in a variety of ways. In general, the bacterial species that have evolved in symbiosis with the pig are known as autochthonous or resident bacteria, which are usually considered “good” indigenous bacteria as they prevent the establishment of allochthonous bacteria [18]. Allochthonous bacteria are those non-resident, non-indigenous bacteria that are “passing through” habitats, representing the opportunistic colonizers that could be associated with diseases or other perturbations [4]. Autochthonous bacteria in a healthy gut represent a climax successional community and a stable equilibrium association with the host, although there are significant site predilections [4,19].

Overall, five of the most common phylogenetic bacteria lineages, as demonstrated through the next-generation sequencing of the variable regions of 16S rRNA genes, are *Eubacterium*, *Clostridium*, *Bacillus-Lactobacillus-Streptococcus* subdivision, *Flexibacter-Cytophaga*-*Bacteroides* group, and *Proteobacteria* phylum [20]. Dowd et al. (2008), also using 16S rRNA gene sequencing technology, identified the top 10 most-frequent genera in the pig gut, which were *Actinobacillus*, *Bacillus*, *Candidatus*, *Clostridium*, *Helicobacter*, *Lactobacillus*, *Ruminococcus*, *Streptococcus*, *Turicibacter*, and *Veillonella* [21]. Isaacson and Kim (2012) reported six major genera: *Clostridium*, *Eubacterium*, *Lactobacillus*, *Peptostreptococcus*, *Propionibacterium*, and *Streptococcus* [4]. A meta-analysis using publicly available 16S rRNA gene sequencing data sets from 20 studies revealed that *Firmicutes*, *Bacteroidota*, and *Proteobacteria* are the core phyla, which totally occupied > 90% of relative abundance among all pig GIT locations, including stomach, duodenum, jejunum, ileum, cecum, and colon [22]. At the genus level, *Clostridium*, *Blautia*, *Lactobacillus*, *Prevotella*, *Ruminococcus*, *Roseburia*, the RC9 gut group, and *Subdoligranulum* were discovered in more than 90% of all gastrointestinal samples [22].

Here, it is worth noting that although bacteria are the predominant microorganisms present in the gut, some fungi, viruses, and protozoa are also present, such as the yeasts of *Kazachstania slooffiae*, *Galactomyces geotrichum*, *Candida catenulate*, and *C. glabrata* [23]. The RNA viruses reported in swine gut include the families of *Picornaviridae*, *Astroviridae*, *Coronaviridae*, and *Caliciviridae*. Also documented are at least two families of DNA viruses, *Circoviridae* and *Parvoviridae* [24]. Healthy pigs typically have fewer viruses in their fecal samples than pigs exhibiting diarrhea [24].

### 2.1. Dynamics of Gut Microbiota at Different Growth Stages of Pigs

As in any ecological community, the gut microbiota of pigs also undergoes significant changes along their growth stage, and relatively, there is a predictable pattern of colonization from the sterile gut of young pigs to climax communities in adult pigs [8,25]. The succession of microbial community in the gut occurs rapidly as a young pig ages, and a myriad of bacteria from the mother, diet, and environment colonize the GIT over time [26]. Following the birth of baby pigs, the gut microbial species are acquired from the environment, particularly from the sow’s milk/colostrum, skin, and feces [27,28]. *Firmicutes* and *Proteobacteria* are the predominant phyla on day 1 after birth, while *Bacteroidota* significantly increase, replacing *Proteobacteria* as the second dominant phyla afterwards [27]. At the genus level, *Bacteroides*, *Blautia*, *Dorea*, *Escherichia*, and *Fusobacterium* are abundant before weaning [29]. The weaning process is a critical time for the pig gut microbiota as the pig transitions from highly digestible milk to a solid feed-based diet. During the nursery stage (a very stressful period), the gut microbiota alters dramatically. The abundance of *Bacteroides*, which are able to utilize monosaccharides and oligosaccharides present in milk, significantly reduces, while *Prevotella*, which can degrade hemicelluloses in plant-based diets, gradually increase and becomes the dominant genus [29,30].

Luo et al. (2022) summarized the results from 63 peer-reviewed publications regarding the dynamic shifts of the gut microbiota in pigs at different ages or different production stages [31]. Based on their meta-analysis of the sequences from 16 studies, a dynamic shift at different ages and growth phases was confirmed. In general, *Bacteroides*, *Escherichia*, *Clostridium*, *Lactobacillus*, *Fusobacterium*, and *Prevotella* are dominant in the nursery stage, then *Prevotella* and *Aneriacter* shift to be the predominant genera, with *Fusobacterium*, *Lactobacillus*, and *Miscellaneous* as comparative minors in the post-weaned stage [31]. The microbial structure becomes relatively stable during the growing and finishing stages, and the population of fiber-degrading bacteria (*Prevotella*, *Roseburia*, and *Clostridium*) enlarges. However, a total of 19 bacterial genera, leaded by *Bacteroides*, *Prevotella*, and *Lactobacillus*, were found in more than 90% of pigs across different stages, which were defined as “core” bacteria in healthy guts of pigs [31].

### 2.2. The Microbial Community along the Gut Sections

**Figure 1:**
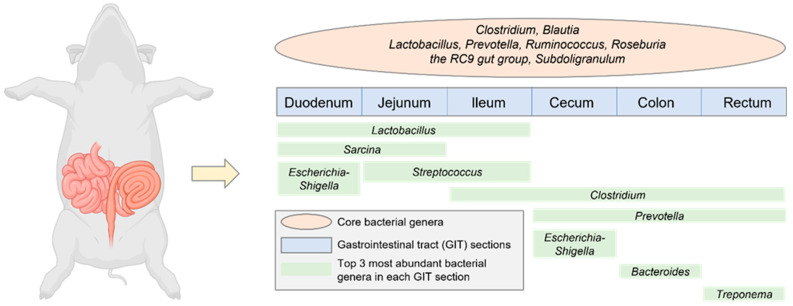
The core and abundant bacteria genera in different sections of pig gastrointestinal tract. Data were adopted from Holman et al. [22] who conducted a meta-analysis using 20 publicly available swine microbiota datasets.

The abundance of core microbial species varies along different sections of the pig GIT (Figure 1). Which bacterial species are predominant in which particular gut section depends on its luminal micro-ecological conditions, such as pH and oxygen concentration, and the available substrates to colonize [8]. Most bacteria in a healthy gut are Gram-positive and obligate anaerobes [32], which thrive in an oxygen-poor environment [8,26]. Nevertheless, there are also a number of Gram-negative bacteria, such as *Bacteroides ruminicola*, *B. uniformis*, *Selenomonas ruminantium*, *Butyrivibrio fibrisolvens*, *Peptostreptococcus productus*, and *Eubacterium riumaerofaciens*, normally present in the cecum [32].

In the areas nearest the stomach, where the pH is low, very few bacteria are present, and those that are present in small numbers are those acid-tolerant species in the genera of *Lactobacillus* and *Streptococcus* [8]. *Lactobacillales*, an order of Gram-positive bacteria, contribute greatly to a healthy microflora on the mucosal surfaces and the maintenance of animal health and growth [33]. *Firmicutes* and *Proteobacteria* are the two predominant phyla in the small intestine, with *Firmicutes* accounting for >70% of the microbiota. *Lactobacillus* is a dominant genus in duodenum (>50%), but its relative abundance decreases to 10~20% in jejunum and ileum, where *Streptococcus* and *Clostridium* are enriched, respectively. As is known, the condition of the ileum is milder (at a nearly neutral pH) than in the duodenum and jejunum.

The AA-fermenting bacteria in the small intestine include *Escherichia* spp., *Klebsiella* spp., *Streptococcus* spp., and others [34]. In the large intestine, Phylum *Bacteroidota* becomes the second largest population (>30%), with *Prevotella* being the most abundant genus [22,35]. The large intestine is also home to large amounts of bacteria capable of fermenting proteins and AA, which include *Bacteroides*, *Clostridium*, *Fusobacterium*, *Peptostreptococcus*, and numerous others [36]. *Lactobacillus* spp. colonize the cecum and colon as well [37,38], provided other obligate anaerobic bacteria in the genera of *Bacteroides*, *Bifidobacterium* and *Clostridium* do not competitively exclude *Lactobacillus* [37,39,40].

To evaluate the phylogenetic composition of the bacterial communities among different intestinal sections, Quan et al. (2018) performed an operational taxonomic unit (OTU) analysis of the microbiota in the ileum, cecum, and colon of Duroc × (Landrace × Yorkshire) pigs [19]. One of the major findings of Quan et al. (2018) was that the numbers of OTUs increased from the small to large intestine: ileum < cecum < colon (522.8 < 916.8 < 994.8 on average) [19]. In terms of taxonomic distribution, at the phylum level, *Firmicutes* constituted 56.0, 37.7, and 62.5%, *Proteobacteria* constituted 41.2, 9.3, and 2.8%, and *Bacteroidota* accounted for 1.3, 46.4, and 29.2% in the ileum, cecum, and colon, respectively. *Fusobacteria* (2.28%) and *Verrucomicrobia* (1.06%) in the cecum, and *Spirochaetes* (2.80%) in the colon were also observed. The most prevalent genera were: *Escherichia-Shigella* (23.1%), *Terrisporobacter* (17.9%), *Romboutsia* (13.7%), and *Clostridium sensu stricto 1* (12.9%) in the ileum, *Alloprevotella* (7.2%), *Lactobacillus* (5.0%), and *Prevotellaceae NK3B31 group* (4.4%) in the cecum, and *Streptococcus* (10.4%), *Lactobacillus* (8.8%), and *Clostridium* (8.0%) in the colon [19].

## 3. Roles of Gut Microbiota in Swine Nutrition and Health

**Figure 2:**
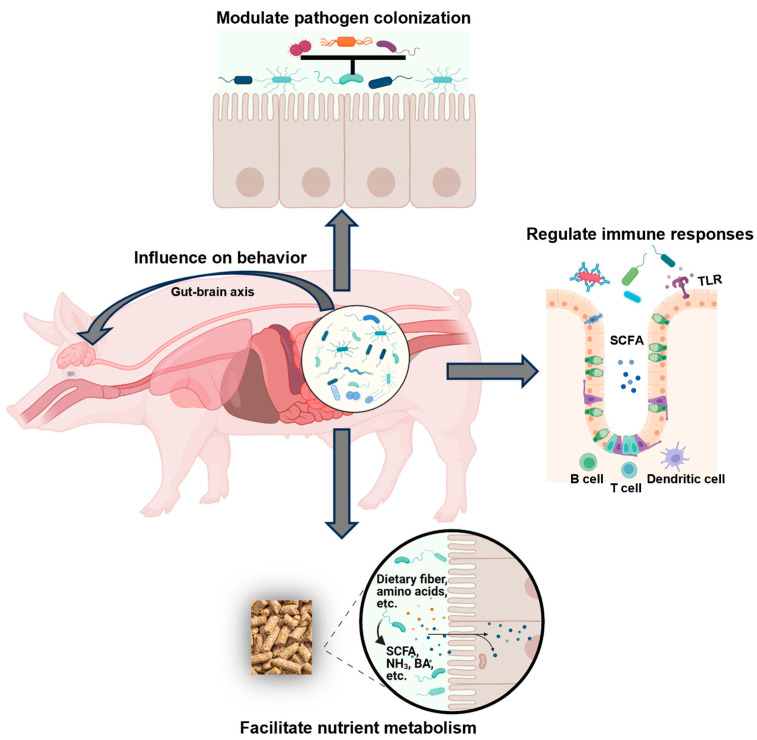
The roles of gut microbiota in swine nutrition, health, and behavior. BA = biogenic amines; NH_3_ = ammonia; SCFA = short chain fatty acids; TLR = Toll-like receptors.

Many researchers now consider the gut microbiota as an endogenous organ of the host, which is required to modulate not only the gut anatomy and physiology but also the health status and functions of the gut, as well as the host’s overall life process [41,42]. In pigs, it is also progressively realized that the diverse nature of gut microbiota, especially those mutualistic microbes, provides numerous benefits related to pig gut structure, health, and functionality [13]. More specifically speaking, it is the diverse metabolic activities and metabolites of the microbiota that largely influences swine nutrition, health, and performance, although some competitive or negative effects from some microbes also exist at the same time [43,44]. As shown in Figure 2, the gut microbiota, in general, supports host life through facilitating nutrient metabolism, immunity regulation, and colonization resistance against pathogens via competition for nutrients and adhesion sites, or the production of antimicrobial substances [44].

### 3.1. Roles on Nutrient Metabolism

Most microbial genes sequenced to date are associated with functions in carbohydrate metabolism, highlighting the integral role of the microbiota in allowing the host to obtain energy from diets [30,45]. In terms of carbohydrate metabolism, ruminant animals receive substantial attention, as the cellulose breakdown in the rumen is not possible by mammalian enzymes. The rumen contains a myriad of bacteria, protozoa, and fungi that produce cellulolytic enzymes needed to break the β 1-4 glycosidic bonds between the glucose monomers contained in the cellulose molecules (also known as fibers) of plant cell walls. This process is called fermentation.

As a non-ruminant species, the pig can only break down fibers via its hindgut (cecum and colon) fermentation with the aid of cellulolytic enzymes from the hindgut microbiota [14], and the fermentation products are short-chain fatty acids (**SCFA**). The most important SCFA are acetate, propionate, and butyrate, which, also called volatile fatty acids (**VFA**), are rapidly absorbed, making them an important energy source for pigs (Grieshop et al., 2001) [46]. Although microorganisms are a major protein source for ruminants, the same is not likely to be the case for pigs because pigs do not have much microbial protein in the small intestine and the microbial protein, or AA, in the colon cannot be absorbed.

A major benefit of the microbial metabolism of dietary nutrients results from the digesta fermentation to release VFA and some vitamins [8,9,47]. Notably, there are many strains of *Fibrobacteres* that were isolated from the pig hindgut and feces [48]. It is known that *Fibrobacteres* species, generally found in the gut of termites and ants, can be found in the rumen of ruminants, where they digest cellulose to produce SCFA in a strictly anaerobic environment [48]. Furthermore, the species in *Bacteroidota* mainly produce acetic and propionic acids, whereas the primary terminal metabolic product of *Firmicutes* is butyric acid [49].

Dietary AA can be metabolized by the luminal microbes to produce microbial metabolites, such as microbial proteins [50]. A number of protein- and AA-fermenting bacteria are present in the large intestine [36]. Dai et al. [51] reported that some microbes, including *E. coli* and *Klebsiella* spp., in the lower gut are highly capable of utilizing Glu, Lys, Arg, and Thr. Luminal Trp that passes through the small intestine is readily metabolizable by some species present in the colon [52]. Fermentation of some AA would also result in the production of VFA that could cross the intestinal wall for further fatty acid synthesis or gluconeogenesis to provide additional energy for the pig. Trp and Thr both have gluconeogenic and ketogenic abilities and can be used in host intermediary metabolism to produce fatty acids or energy, whereas Lys has ketogenic ability only, and Arg and Gln are strictly glucogenic. Nevertheless, being able to utilize these AA in gluconeogenesis, ketogenesis, or both highlights the importance of gut microbiota to readily use AA to support the metabolic demands of pigs.

Several species of intestinal bacteria, such as *Lactococcus lactis* subsp. *cremoris* L. *lactis* subsp. *lactis, Lactobacillus plantarum, Bacteroides, Streptococcus thermophilus*, *E. coli* K-12, *Morganella morganii, Klebsiella pneumoniae, Hafnia alvei*, and *Clostridium*, all have tryptophanase for Trp catabolism [53] to maintain bacterial growth and survival [54,55,56]. Some human and mice studies showed three Trp metabolic pathways leading to serotonin, kynurenine, and indole derivatives in the gut, which are under the direct or indirect control of the microbiota [55].

The gut microbiota is known to synthesize vitamins, especially vitamin B and K. Phylum *Bacteroidota* is predicted to contain the largest number of vitamin B producers based on microbial genomic analysis [57]. Some bacteria produce menaquinones and vitamin K quinones. The fecal vitamin K content is strongly associated with *Prevotella* spp. (MK-5, -11, -12, and -13), *Bacteroides* spp. (MK-9 and -10), and *Escherichia*/*Shigella* spp. (MK-8) [58]. In addition to VFA release and vitamin syntheses, the microbiota also regulates the metabolism of many other nutrients (including glycolipids and AA) and bile acids [59,60,61,62]. Research has already shown that those resident microbial species are more crucial to host metabolic homeostasis and health [63].

The utilization of AA by gut microbiota plays an important role in dietary nutrient digestion and metabolism, which can reciprocally influence the gut microbial population, gut tissue health, and gut physiological functions [64,65,66,67]. It is important also to note that AA metabolism by the microorganisms can come at a cost to the host, as the microorganisms are competing against the host for nitrogen, and the microbial metabolism may produce toxic metabolites for the host as well [62]. Unlike the fermentation of carbohydrates, the fermentation of protein and AA can result in a number of additional metabolites such as branched chain fatty acids, but also some potential toxic products including ammonia, amines, phenols, and indoles [44,50]. Certainly, more work is needed to understand what sort of metabolic balance is optimal to support not only the microbial metabolism but also the host health and feed efficiency [14].

### 3.2. Modulation of Pathogen Colonization

As the first tier of defense, a healthy gut microbiota confers benefits by indigenous species to the pig by competitively excluding the colonization of nonindigenous species that could be pathogenic [8]. The indigenous species could prevent colonization by nonindigenous species through direct competition for nutrients or mucosal attachment sites (mucus or epithelial surface), or through alteration of the local growth environment via the production of antimicrobial compounds, VFA, and chemically modified bile acids [68].

Recently, there has been significant interest in the concept of competitive exclusion as a potential measure for preventing intestinal diseases in livestock species. The applied practice consists of providing newborn animals with oral supplements of either defined or undefined mixed bacterial cultures in order to outcompete pathogenic bacteria suggested to underlie colonization resistance [69]. The effectiveness of this competitive exclusion strategy is likely the most efficacious in animal herds carrying significant pathogen loads. However, in clean or non-infected herds, it may lead to potential growth costs due to intestinal responses to bacterial colonization [70]. Although the proposed mechanisms for competitive exclusion have included bacterial interference, bacterial antagonism, and colonization resistance, generally any specific mechanisms have not been fully defined [43].

### 3.3. Regulation of Immune Function

The gut microbiota can also confer benefits to the pig by directly stimulating the gut immune processes [8]. In general, the spore-forming *Clostridium* strains are recognized as the primary cause of intestinal disorders or diarrhea in neonatal and newly weaned pigs [71,72]. Previous studies reported that it was the spores and toxins that originated from *C. difficile* and *C. perfringens* that induced high rates of diarrhea and mortality after weaning [72,73]. Moreover, *Terrisporobacter* produces a uremic toxin called trimethylamine-*N*-oxide, which is associated with oxidative stress and inflammation in the gut of weaned pigs [74]. However, the gut microbiota of healthy piglets is different from that of the diarrheal ones, which are more diverse and harbor more abundant beneficial bacteria like *Lactobacillus* that inhibit pathogen colonization [75]. As is also known, pathogenic bacteria can be recognized by Toll-like receptors (TLRs) that play a critical role in host innate immunity [76].

The commensal bacteria in the gut can promote the differentiation of immune cells, such as the regulatory T cells [77]. Moreover, the SCFA produced by gut microbial fermentation can also regulate host immune responses, such as by promoting the anti-inflammatory properties of colon dendritic cells 128]. Butyrate can enhance the intestinal barrier, and acetate can mediate a microbiome–brain–β-cell axis to ameliorate metabolic syndrome [78,79]. Species in the *Ruminococcaceae*_UCG-002 genera can produce butyrate, which plays a key role in colon health [80,81]. *Actinobacteria*, as Gram-positive filamentous bacteria, can serve as sources of novel antibiotics and secondary metabolites that may be used in medicine or to improve the disease resistance and growth performance of animals [82].

### 3.4. Microbial Influences on Swine Behavior

Gut microbiota also plays a role in swine behavior, particularly affecting their stress response and appetite or feeding behavior [83]. He et al. (2022) reported in three breed groups an interaction between gut microbial composition and feeding behavior of swine and identified multiple bacteria (e.g., *Lachnospiraceae*, *Blautia* and *Ruminococcaceae*_UCG-014) that were associated with feeder occupation time, feeding rate, number of visits to the feeder, as well as feed intake [84]. These bacteria are known to produce SCFA that is essential in regulating appetite through the microbiome-gut-brain axis [85].

Early weaned piglets are typically exposed to psychosocial and environmental stressors, including diet change, separation from mothers, and alteration of housing. Stress and the hypothalamus-pituitary-adrenal (HPA) axis, which regulates stress responses, are reported having interactions with gut microbiota [86]. The HPA axis in response to psychological stress can lead to a leaky gut and adherence of pathogenic bacteria, such as *Salmonella* [87], which boosts the pro-inflammatory cytokines in the bloodstream [88] and results in gut microbial dysbiosis.

Pigs with a diverse gut microbiota have been found to exhibit a lower stress response compared to those with a less diverse microbiota [89]. SCFA (Trp metabolites), especially butyrate, have been shown to affect catecholamine pathways, influencing the synthesis of neurotransmitters dopamine, norepinephrine and epinephrine, so there must be some links between the gut microbial metabolism of Trp and the secretion of anorectic gut hormones, such as glucagon-like peptide-1 and peptide YY [90]. Gao et al. (2019a) reported that AA metabolism in gut microbiota may have an important regulatory relationship in mediating the communication or crosstalk between the microbes and the host brain, owing to the synthesis of hypothalamic neurotransmitters by the microbes in response to aromatic AA [91]. Moreover, SCFA can also modulate the gut–brain axis by affecting neuroinflammation and neuronal function. For example, butyrate has been shown to promote an anti-inflammatory phenotype in microglia, reducing the production of pro-inflammatory cytokines and neuroinflammatory responses [92].

In short, research has suggested that there is definitely an interplay between the gut and behavior through various pathways. The symbiotic relationships between the gut microbiota and its host contribute to the overall health, welfare, behavior, and production performance of pigs and, thus, are particularly important in swine production [14,93,94].

## 4. Effects of Dietary Nutrients and Feed Additives on Swine Gut Microbial Composition

Many factors that can be defined as microbiota modulators can alter the composition of gut microbiota in swine [45,95,96]. Payen et al. (2023) recently reviewed the effects of several families of modulators on the gut microbiota of swine and their consequences on host physiology [97]. Diet, especially the ingredient composition or the nutrient plane, is fundamentally important to determine the gut microbial composition [15,62,98]. Changes in diet composition that affect the ratios of soluble to insoluble carbohydrates alter digestion rates [99], as well as the ileal and cecal microbiome by selectively promoting the growth of certain bacteria [100]. For example, feeding potato starch can alter the hindgut microbiota by increasing the abundance of some species (e.g., *Turicibacter* and *Ruminococcus*) and decreasing the abundance of others, such as *Clostridium* [101]. Other effects of changing dietary substrates (with beet pulp additives and rice diets) include shifts in microbial communities, which were thought to be an adaptation to enhance the digestion of the changed substrates [20,39,102].

Autochthonous bacteria are adapted to grow in specific gut conditions, which include pH level, oxygen level, and health status, as well as diet and other environmental and social conditions [4,44]. *Prevotella* in the gut microbiota was more common in the weaned pigs than in the breast-feeding piglets because *Prevotella* could ferment the indigestible polysaccharides into SCFA in the gut [103]. In addition, *Anaerovibrio lipolytica* was linked to fat metabolism through the production of lipase to hydrolyze triglycerides [104].

Dietary use of antibiotics was a common practice (in the past) to enhance the growth performance of pigs, and doing so can increase the growth rate by 16.4% and feed efficiency by nearly 7% [105]. The mechanism by which growth is promoted by antibiotics is still unclear, but it may be related to how they alter the gut microbial composition [4,106,107]. When the composition of bacteria changes, the number of bacteriophages sometimes also changes as a secondary effect of antibiotic administration [108]. Additionally, provisions of probiotics, prebiotics, essential oils, milk replacers, and other feed additives have been shown to have regulating effects on the gut microbiota in swine and, especially, in young piglets [62,69,109,110,111,112]. Feeding pigs with *Bifidobacterium breve* as a probiotic can even alter the fatty acid composition of their adipose tissue, but more work is still needed to understand the mechanistic links between gut microbiome and lipid metabolism [62,113].

## 5. Effects of Dietary Amino Acids on Swine Gut Microbial Composition and Metabolism

As is known, the dietary protein and AA supply can be readily manipulated by altering the amount of crystalline AA added, and the AA profile of a diet can have a profound impact on gut microbial composition, gut health, and thus gut functionality [13,50,114,115,116]. Although a few mechanisms through which dietary AA acts on gut microbiota have been recognized [26], the particular mechanisms regarding particular AA affecting particular microbial species in swine guts are still not clear.

Numerous studies (Supplemental Table S2) have shown how the microbial composition and diversity in swine gut can be altered by dietary AA supply [117,118,119,120]. The study by Zhou et al. (2020) demonstrated that an optimal AA profile in an antibiotic-free, low-protein (**LP**) diet can efficiently improve the gut health and growth performance of weaned pigs through optimizing the gut microbial structure, reducing the gut permeability, and lowering the plasma endotoxin concentration [121]. Zhao et al. (2020) also reported that dietary protein level and essential AA pattern both altered the structural composition of the colonic microbiota in barrows [116]. Liu et al. (2023b) reported the effects of LP diets with balanced four essential AA (Lys, Met, Thr, and Trp) on the cecal microbial composition of finishing pigs [122]. The relative abundance of *Turicibacter*, *Terrisporobacter*, *Clostridium_sensu_stricto*_1 and UCG-005 was higher, while the abundance of *Lactobacillus* and *Streptococcus* was lower in pigs fed with the LP diet compared with the normal protein diet. The content of tyramine, spermidine and histamine were negatively correlated with the abundance of *Terrisporobacter*, and the content of histamine was positively correlated with the abundance of *Lactobacillus*. The results indicated that a decrease in dietary protein can change the profile of the cecal microbiota and reduce the content of cecal bioamine [122].

That said, Lee et al. (2023) reported that their LP diets with different crystalline AA supplementation patterns did not affect the bacterial diversity in the colon digesta of the weaned pigs [123]. Zhao et al. (2020) observed no differences in the alpha-diversity of the colonic microbiota in fattening pigs fed diets with different dietary protein levels or crystalline AA-provided patterns [116]. The “inconsistent” results obtained from these studies may be explained by their experimental designs that involve animal differences, the amount and source of dietary fermentable carbohydrates, and the sampling time.

### 5.1. Tryptophan (Trp)

Some metabolites of Trp can modify intestinal microbial metabolism, microbial composition, and the host–microbiome interface [80]. Dietary Trp supplementation improves the growth of weaning pigs and regulates the composition of their hindgut microbiota [124]. The alpha diversity indices were enhanced in response to Trp supplementation in weaned piglets [124] and fattening pigs susceptible to intestinal adhesion of enterotoxigenic *Escherichia coli* (ETEC) F4 [125]. In the experiment with piglets, dietary Trp supplementation (0.2% to 0.4%) markedly altered the intestinal microbial composition as evidenced by enhanced alpha and beta diversity in the microbiome [124]. Trp supplementation was also associated with the increased abundances of *Prevotella*, *Roseburia*, and *Succinivibrio* genera, and with the reduced abundances of opportunistic pathogens, such as *Clostridium sensu stricto* and *Clostridium* XI in the cecum [124]. The reduction of *Clostridium* species indicated an inhibitory effect of Trp or its metabolites on potential intestinal pathogens [55,126,127]. It is reported that *Prevotella* and *Roseburia*, belong to *Bacteroides* and *Firmicutes*, respectively, produce SCFA, critical molecules with the ability to regulate intestinal homeostasis in humans and animals [128,129,130]. In line with these reports, Trp supplementation increased the concentrations of SCFA in the large intestine of weaned pigs [124].

In another study on weaned pigs, 0.4% Trp supplementation for 4 weeks increased *Lactobacillus* and *Clostridium XI* in the jejunum [131]. The abundances of *Clostridium sensu stricto* and *Streptococcus* (two opportunistic pathogens) were reduced by the 0.2% to 0.4% dietary Trp supplementation [131]. The metabolites produced by several bacterial species [53] can benefit the host by regulating the intestinal microbial diversity [80]. Of note, the regulatory effect of Trp on Trp-metabolizing bacteria was observed in the jejunum instead of the hindgut [131], indicating a different response of different segments of the GIT to dietary Trp supplementation. The exact reason for this phenomenon remains unknown. It is possible that supplemental Trp does not enter the large intestine of pigs because it is both absorbed into enterocytes and utilized by bacteria in the small intestine. It is also possible that the small intestine of piglets might be a suitable environment for the survival and colonization of Trp-metabolizing bacteria. Trp supplementation (at 0.2%) to lipopolysaccharide (LPS)-challenged pigs increased the relative abundance of *Anaerostipes* while decreasing the abundance of *Corynebacterium* and unclassified_c_*Bacteroidia* in the colon [132]. The data implied that Trp helped in maintaining colonic mucosal microbiota homeostasis in LPS-challenged piglets by supporting beneficial bacteria colonization and inhibiting pathogenic bacteria [124,126].

### 5.2. Arginine (Arg)

Arg can modulate AA utilization in the pure culture of bacteria or mixed bacteria obtained from porcine intestines [51]. In an in vitro study based on pure bacterial strains and mixed bacterial cultures derived from the intestinal content of piglets, Dai et al. [133] reported that Arg can significantly influence, in a species- and gut tract-dependent manner, the bacterial metabolism of the Arg-family of AA and also the Ser- and Asp-family of AA and the utilization of most AA since Arg can be used as a nitrogen source for the small-intestinal bacteria. Thus, the metabolism of Arg by small-intestinal bacteria not only plays a crucial role in the growth of the bacteria but is also regarded as a surviving strategy for their colonization in the small intestine [117].

He et al. (2011) reported that the dietary addition of Arg was not able to restore the disturbed gut microbiota, although it alleviated the weaning stress in the piglets [134]. Luise et al. (2020) showed that dietary Arg supplementation did not influence the fecal microbial structures in sows, which suggested that Arg did not affect the sow’s intestinal eubiosis, and as a consequence, it can be assumed that it did not affect the environmental microbiome where the new-born piglets were born and raised [135]. Nevertheless, although the microbial structure was not profoundly affected, some taxa that are common in the sow intestine were influenced by Arg supplementation. The Arg supplementation increased both the *Bacteroides* genus and the *Bacteroidaceae* family in feces. This result is in accordance with a previous study by Wu et al. (2011), in which a higher abundance of this bacterial family was associated with a human diet with high levels of animal proteins, suggesting that this bacterial family can use protein and AA for its metabolism [136]. In addition, Arg supplementation reduced several bacterial families (*Succinivibrionaceae*, *Acidaminococcaceae*, *Veillonellaceae*) and *Succinivibrio* genus [135]. The reduced abundance of *Succinivibrio* in the Arg group could be associated with the limited use of Arg from these bacteria, as suggested by Dai et al. (2010) for *Succinivibrio dextrinosolvens* [34]. In fattening pigs, 1.0% Arg supplementation for 60 days increased *Cyanobacteria*; and in combination with 1.0% Leucine (Leu) it increased *Bacteroides* and reduced *Clostridium sensu stricto*, *Terrisporobacter* and *Escherichia-Shigella* in the colon [137]. Thus, it was concluded that Arg supplementation should be beneficial for maintaining gut health and functions in neonatal piglets.

Arg can regulate nitrogen recycling in the gut to benefit the nutrition and health of the organisms. It is known that nitric oxide (NO) produced from Arg by intestinal mucosal cells can kill pathogenic bacteria [138,139]. Therefore, Arg metabolism and the production of corresponding metabolites by the luminal bacteria might reduce Arg availability for NO synthesis and regulate the metabolism of Arg-family AA in small intestinal mucosal cells, thereby indirectly affecting NO synthesis. Van den Abbeele et al. (2022) reported that Arg and Lys specifically increased the propionate level, likely produced by *Muribaculaceae* members. So the selective use of AA by gut microbes can produce health-related SCFA, thus confirming the prebiotic potential of specific functional AA [140]. In addition, Matsumoto et al. (2019) reported that the release of polyamines after Arg utilization by intestinal bacteria may help enhance endothelial function in humans [141].

### 5.3. Aspartate (Asp)

D-Asp, found in the cell walls of some Gram-positive bacteria, such as *Lactococcus lactis*, *Enterococcus faecium*, *Lactobacillus fermenti*, and *Streptococcus faecalis*, regulates bacterial cell wall growth and remodeling. Dietary supplementation of L-Asp at 1% enhanced bacterial diversity (Shannon and Simpson indices). Dietary DL-Asp at 1% also increased the Simpson index compared with the control group. However, D-Asp failed to influence gut microbial evenness, richness, and diversity in the terminal ileum [142]. Compared with the control group, D-Asp markedly decreased *Actinobacteria* abundance. However, L-Asp increased *Nitrospirae*, *Gemmatimonadetes*, *Acidobacteria*, and *Chlorobi* abundance, whereas it decreased *Tenericutes* abundance. Also, *Proteobacteria* and *Actinobacteria* abundances were reduced in the DL-Asp group. At the genus level, the abundances of *Lactobacillus*, *Weissella*, *Pediococcus* and *Streptococcus* were decreased, whereas the abundances of *Idiomarina* in the L-Asp group were increased [142].

It was further found that dietary L-Asp supplementation at 1% enhanced the intestinal abundances of *Actinobacteria* and *Bacteroidota* but decreased that of *Firmicutes* at the phylum level and that of *Sphingomonas* and *Massilia* at the genus level [142]. *Actinobacteria* show antiviral activity against pathogens [143,144]. An increase in *Firmicutes* but a decrease in *Bacteroidota* in the DL-Asp group were also observed. It was reported that an obese human had a higher level of *Firmimicutes* and a lower level of *Bacteroidota*, suggesting that DL-Asp may induce fat deposition associated with growth performance [145]. Meanwhile, D-Asp markedly increased the abundances of the phylum *Tenericutes* and that of *Clostridium sensu stricto 1*, *Streptococcus*, and *Intestinibacter* at the genus level. The proportion of *Clostridium sensu stricto 1* in finishing pigs was significantly decreased with a reduction in dietary protein level [146]. In addition, *Escherichia-Shigella* exists widely in patients with inflammatory bowel diseases [147]. In the study by Li et al. (2019), L- and DL-Asp reduced the *Escherichia-Shigella* abundance, whereas D-Asp increased the abundance, suggesting 1% D-Asp may induce intestinal inflammation [142].

Supplementation with *N*-carbamoylaspartic acid (NCA) in sows significantly increased the abundance of *Bacteroidota* and reduced the abundance of *Firmicutes*, the ratio of *Firmicutes* to *Bacteroidota*, *Melainabacteria*, and *Kiritimatiellaeota* phyla in feces at day 113 of gestation [148]. The abundance of *Cellulosilyticum*, *Fournierella*, *Anaerovibrio*, and *Oribacterium* genera was reduced by NCA. In addition, maternal supplementation with NCA significantly enriched the abundance of *Catenisphaera* and reduced the abundance of *Lachnospire*, *Faecalibacterium* and *Anaerovorax* genera on the 14^th^ day of lactation [148]. It was suggested that maternal supplementation with NCA mainly regulates the utilization of lipid and carbohydrate by regulating the abundance of specific gut microbes, which may contribute to decreased backfat loss in sows during lactation and a heavier birth weight in piglets after NCA treatment [148].

### 5.4. Glutamate (Glu) and Glutamine (Gln)

Gln and Glu specifically stimulated acetate and butyrate production, relating to the stimulation of a range of families containing some known butyrate-producing species in *Ruminococcaceae*, *Oscillospiraceae*, and *Christensenellaceae* families [140]. The abundance of *Bacteroidota* and *Peptostreptococcus* in pig ileum was also increased by dietary Glu supplementation. At the phylum level, Glu increased the *Actinobacteriota* abundance and the *Firmicutes*/*Bacteroidota* ratio while decreasing the *Firmicutes* abundance. At the genus level, Glu improved the abundance of beneficial bacteria (e.g., *Lactobacillus*, *Prevotellaceae*-NK3B31 group, and UCG-005) in the colon [149]. Feng et al. (2015) also reported that Glu (in a monosodium form) can markedly change the composition of, and increase the diversity of the gut microbiota in growing pigs by promoting the colonization of *Faecalibacterium prausnitzii* and *Roseburia* [150]. This is consistent with the important roles of Glu in regulating the nitrogen balance in bacteria [117]. The relative abundances of *Prevotella* and *Anaerovibrio* were also higher in the gut of pigs fed a typical weaner diet supplemented with 0.5% Glu [151]. The weaned pigs fed Glu had less *Clostridium* and *Terrisporobacter* (genera) in the gut [151], and it is known that *Clostridium* are recognized as the primary cause of diarrhea in neonatal and weaned piglets [72]. *Terrisporobacter* is associated with oxidative stress and inflammation in the gut of weaned pigs [74]. Therefore, it is plausible that the increased abundances of *Prevotella* and *Anaerovibrio* in the gut of weaned pigs fed Glu may improve their gut health by stabilizing the intestinal environment and immune state of weaned pigs via the reduced abundances of *Clostridium* and *Terrisporobacter*.

Dietary Gln supplementation at an earlier age in piglets may yield better beneficial effects on their gut microbiota. Yan et al. (2019) reported that dietary glycyl-glutamine (Gly-Gln) supplementation (at 0.25%) significantly shifted the piglets’ gut microbiota during the weaning transition [152]. The 16S rDNA high-throughput sequencing analysis revealed that the Gly-Gln supplementation increased gut bacterial loading, elevated alpha diversity, and increased the relative abundance of anaerobes and fiber-degrading bacteria (Phylum *Fibrobacteres*) in a time-dependent manner. The Gly-Gln supplementation increased the relative abundance of *Fibrobacteres* and *Bacteroidota*, but decreased that of *Firmicutes*, in the gut of piglets on day 38 [152]. Consistent with this, Zhang et al. (2017) reported that the late-gestation sows suffering from constipation may be treated and relieved by dietary Gln supplementation (at 1.0%) because Gln supplementation can regulate the intestinal microbial composition by markedly increasing the abundance of intestinal-friendly bacteria (e.g., *Bacteroidota*) [153].

Glu at 1% significantly increased the concentrations of SCFA in the colonic contents of piglets [149]. The SCFA produced by the gut microbiota can enhance the intestinal barrier [78], so the increased abundance of *Fibrobacteres* may contribute to the Gly-Gln’s beneficial effect on weaning piglets. Gly-Gln supplementation was also reported to enrich the SCFA-producing bacteria, including *Butyricicoccus pullicaecorum* [154], *Faecalibacterium prausnitzii* [155,156], and *Oscillibacter valericigenes* [157]. Collectively, dietary Gly-Gln supplementation can improve the gut microbiota in piglets and increase the concentrations of SCFA in gut digesta.

Zeng et al. (2015) reported that dietary N-carbamylglutamate supplementation increased the growth of cecal *Lactobacillus* spp. and anaerobic bacteria in neonatal piglets [158]. This occurred probably because *N*-carbamylglutamate regulated the synthesis of Arg in the intestine [159], and *Lactobacillus* and anaerobic bacteria in the cecum could utilize Arg [133]. It was also reported that *N*-carbamylglutamate supplementation influenced the fecal microbial community structure of pregnant sows subjected to fixed-time artificial insemination to a certain extent, and it can improve both the number of piglets born alive and the uniformity of piglets’ birth weight [160]. Supplementation of mixed doses of Glu and Gln could favor the growth of AA-fermenting bacteria, such as *Enterococcus*, *Pediococcus* and *Selenomonas*, in the large intestine of piglets without compromising the gut microbial ecosystem after 3 weeks [161].

### 5.5. Sulfur-Containing Amino Acids (SAA)

Dietary supplementation of cysteine (Cys; a functional SAA) can also shift the composition of intestinal microbiota in pigs. Xu et al. (2014) reported that dietary *N*-acetyl Cys (**NAC**) supplementation increased the *Lactobacillus* and *Bifidobacterium* counts, while decreasing the *Escherichia coli* count in the intestinal content of weaned piglets [162]. Additionally, the supplementation of NAC is promising in protecting piglets from the microbial dysbiosis caused by porcine epidemic diarrhea virus infection. NAC supplementation increased the abundance of *Lactobacillus* in both the healthy and the porcine epidemic diarrhea virus-infected piglets [98]. Furthermore, Luo et al. (2019) reported that NAC supplementation altered the fecal microbial communities of the sows at their late gestation stage [163], which was consistent with the previous study in weaned piglets [162], and the changes in fecal microbiota were positively correlated with nutrient transport, which could affect maternal metabolism.

The genus of *Lactobacillus* is an important beneficial bacterium in the gut that can prevent gastrointestinal infection. Valeriano et al. (2017) and Ding et al. (2019) both reported that a high intake of Cys decreased the abundance of *Lactobacillus* [21,164]. Maternal intake of 0.5% Cys significantly increased the relative abundance of *Proteobacteria* (phylum) in the jejunum and ileum of the piglets [165]. As is known, *Proteobacteria* consist of a variety of pathogens, such as *Escherichia*, *Salmonella*, *Vibrio*, and *Helicobacter*. The expansion of *Proteobacteria* is associated with the pathogenesis of inflammatory bowel disease (IBD), especially those with adherent and invasive properties, which might drive proinflammatory changes and eventually lead to an IBD development [166].

In addition, maternal intake of 0.5% Cys increased the level of *Bacteroidota* phylum and *Bacteroides* genus and decreased *Firmicutes* phylum in the cecum and colon of piglets compared with the 0.3% Cys group [165]. In a study with sows in late pregnancy, 0.4% Cys supplementation increased the abundance of *Bacteroidota* in feces compared with 0.5% Cys [21]. Similar microbial changes were also reported in children. De Filippo et al. (2010) found that African children had a significant enrichment in *Bacteroidota* and depletion in *Firmicutes*, whereas European children had a lower abundance of *Bacteroidota* and a higher abundance of *Firmicutes* and obesity [167]. Based on these results, a hypothesis was made that a lower ratio of *Firmicutes*/*Bacteroidota* is more helpful for digesting the polysaccharide-rich diets and defending against intestinal inflammation and colonic diseases. In contrast, a higher *Firmicutes*/*Bacteroidota* ratio is usually induced by a diet rich in fat and sugar and poor in fiber, ultimately resulting in obesity. Thus, the decreased *Firmicutes*/*Bacteroidota* ratio in suckling piglets induced by maternal intake of 0.4% and 0.5% Cys may result in less body fat mass [165]. The 0.4% Cys improved fecal microbial diversity compared with the 0.5% Cys in sows. The 0.4% Cys group showed increased abundance of *Ruminococcaceae*_UCG-002 and *Prevotellaceae*_NK3B31_group, but decreased the abundance of *Lactobacillus* and *Pseudobutyrivibrio*, whereas the 0.5% Cys group had decreased abundance of *Lactobacillus* [21].

A higher proportion of maternal SAA supplementation (62% Met in 0.78% total SAA) also increased the concentrations of *Proteobacteria* in the piglet’s colon and cecum. The 51% Met supplementation group had a higher relative abundance of *Firmicutes* [168]. These results indicated that a diet consisting of 51% Met is an optimum Met to Cys ratio for sows from late pregnancy to lactation to maintain offspring health by improving the serum biochemistry, altering the plasma metabolomics profile, and altering the intestinal microbiota composition, whereas a high Met to Cys ratio may increase the possible risk to offspring health. Furthermore, trials have shown that adding 0.48% Met to lactating sows’ diets can increase the abundance of *Phascolarctobacterium* and *Bacteroides*, contributing to piglet health [169].

### 5.6. Branched-Chain Amino Acids (BCAA)

The impact of AA on a piglet-derived colonic microbiota was evaluated using a 48 h *in vitro* batch incubation strategy. BCAA (Leu, Ile, and Val) strongly increased branched-chain fatty acids and valerate levels, which coincided with a marked increase in *Peptostreptococcaceae* [140]. Yang et al. (2016) studied the effects of dietary supplementation of a BCAA mixture on the gut microbiota in middle-aged mice, found that BCAA can influence the gut microbial diversity, and concluded that dietary BCAA supplementation may improve pig metabolism and health [170]. The colon content of pigs offered a mixture of Val above and Ile at the NRC (2012) levels had a higher abundance of *Actinobacteria*, *Enterococcus*, and *Brevibacillus*, and that with Val above the NRC (2012) level was more enriched with *Mogibacterium* [171]. It appears that the improvement in growth performance of pigs fed with Ile and added Val might be due to the benefits of their highly abundant colonic bacteria [172]. *Firmicutes* were the most abundant in the colon of the Leu group in finishing pigs [137], which is consistent with previous findings of elevated *Firmicutes* abundance and reduced *Bacteroidota* abundance in obese mice [173] and obese humans [174]. The abundance of *Actinobacteria* in the colonic contents of Duroc × Large White × Landrace finishing pigs was the highest in the dietary Leu supplementation group [137], which is in line with the report of Pedersen et al. (2013) [175], who observed a higher *Actinobacteria* abundance in the cecal microbiota of obese Göttingen minipigs. In a piglet model, Yin et al. (2020) found that balanced BCAA markedly improved the proliferation of *Lactobacillales* and *Aeromonadales*, and they concluded that BCAA, especially Leu and Val, when balanced appropriately, can have a significant role in mitigating the negative effects of LP diets on the growth performance of pigs by altering their gut microbial composition [176]. Recently, Spring et al. (2020) also reported that pigs offered a LP + BCAA diet had higher abundance of *Paludibacteraceae* and *Synergistaceae* in their feces, while being less enriched in *Streptococcaceae*, *Oxyphotobacteria* unclassified, *Pseudomonadaceae* and *Shewanellaceae* [177]. This result might be suggestive of better health and carbohydrate digestion capacity in pigs.

### 5.7. Other Amino Acids

*Serine (Ser):* Ser and perhaps Asp may be “essential” for gut bacteria not only by serving as building blocks of cellular components but also by participating in the synthesis of secretory molecules that may be important for bacterial adaptation and colonization in the small intestine, as well as their interactions with the host [178]. Burnside et al. (2010) revealed that the utilization of Ser and Asp in the pathogenic bacteria *Staphylococcus aureus* might be related to the production of phosphopeptides, which contribute to bacterial virulence [179]. The major products from Ser catabolism are pyruvate and ammonia, which could serve as energy and nitrogen sources for bacteria growth [180,181,182,183]. Ser can also be rapidly metabolized by *E. coli* [133]. And the growth of *Streptococcus* sp. or *Klebsiella* sp. was stimulated in the presence of Ser [180,183,184].

*Lysine (Lys):* The extensive catabolism of dietary Lys in the gut is taken care of by the luminal bacteria rather than the enterocytes, and, therefore, it can be postulated that dietary Lys restriction can affect the gut microbiota [66]. Yin et al. (2017) first reported that dietary Lys restriction enhanced the intestinal richness and evenness of the microbial community in piglets [185]. Lys-restricted diet (70% of the control) markedly improved feed intake and inflammatory status via mediating the gut microbiota [186,187]. Higher levels of Phyla *Actinobacteria*, *Saccharibacteria*, and *Synergistetes* were observed in the Lys-restricted group. Another study on the long-term effects of Lys restriction, conducted by the same laboratory [187], reported that the abundances of *Escherichia-Shigella*, *Aquabacterium*, and *Candidatus Methylomirabilis* were enhanced with a 30% Lys limitation during the whole experiment. Dietary Lys restriction reduced the abundances of *Streptococcus*, *Bacteroides*, *Bacillus*, *Pasteurella*, *Clostridium sensu stricto*, *Faecalibacterium*, *Paucisalibacillus*, and *Lachnoclostridium*. The abundance of *Weissella* was decreased during the growing period but enhanced during the adult period in the Lys-restricted group.

*Histidine (His):* Dietary His failed to affect bacterial diversity, but His-treated piglets exhibited higher abundances of *Butyrivibrio* and *Bacteroides* compared with the control and protein-restricted piglets [188]. *Butyrivibrio* has been identified in pigs with high residual feed intake [189], indicating that *Butyrivibrio* may be involved in feed intake regulation. However, the mechanism of His-mediated *Bacteroides* proliferation and its role in piglets have not been illustrated.

*Proline (Pro):* Dietary Pro supplementation (at 1%) decreased the proportion of *Prevotella* in the proximal colonic contents and that of *Klebsiella pneumoniae* and *Peptostreptococcus productus* in the distal colonic contents. As is known, these species of bacteria can metabolize dietary carbohydrates, especially indigestible fiber [136]. The study of Huanjiang mini-pigs by Ji et al. (2018) indicated that dietary Pro supplementation affects the microbial composition as well as their metabolite composition in the colonic lumen [190]. Ji et al. (2018) also reported that dietary Pro supplementation can mediate gut microbial diversity, which may further affect the nutrient metabolism and health of the pig [190].

*Glycine (Gly):* The colon content of piglets fed with 2% Gly exhibited a reduction in abundance of pathogenic bacteria (*Escherichia–Shigella*, *Clostridium*, and *Burkholderiales*) and an increase in SCFA-producing bacteria (*Blautia*, *Lachnospiraceae*, *Anaerostipes*, and *Prevotella*), indicating that dietary Gly elevated the ratio of beneficial to harmful bacteria and may be related to the strengthening of immunologic barrier function [191].

### 5.8. Implications for Swine Nutrition and Health

As reviewed above, the gut microbiota plays a pivotal role in maintaining the overall health and well-being of swine by influencing swine gut structure and functions for nutrient acquisition, immune function, and disease resistance. Research reported in the literature so far has provided valuable knowledge into the compositional changes upon dietary manipulation as well as some not-so-clear microbiota-phenotype relationships, that is, the associations between the gut microbial communities and the production traits of swine.

As is known, AA serves as a substrate for microbial metabolism inside the gut. Understanding the intricate relationship between AA and gut microbiota holds profound implications for swine production practices. Exploring the impact of AA on gut microbiota composition and metabolism can also shed light on how dietary composition can modulate the structure and functions of the gut microbial community. A balanced and optimized AA profile in swine diets promotes a favorable gut microbiota, fostering the growth of beneficial bacteria while inhibiting the proliferation of harmful pathogens. This knowledge allows for the formulation of the best-possible diets that not only meet the nutritional requirements of swine but also enhance their gut health, leading to improved nutrient utilization, disease resilience, and overall productivity. Consequently, a deeper understanding of the interplay between AA and gut microbiota empowers swine nutritionists and producers to implement precision feeding strategies, promoting the economic profitability of pork production as well as the well-being of the animals.

The current global demand for antibiotic-free pork production is an impetus for swine nutritionists and veterinary clinicians to tailor swine diets to positively influence gut microbiota, which can mitigate gastrointestinal disorders and enhance animal disease resistance. In veterinary practice, understanding the AA-microbiota interplay can facilitate the development of preventive and therapeutic strategies. Moreover, this understanding will allow veterinarians to design targeted interventions, such as using “designed” dietary AA composition plus specific probiotic and/or prebiotic additives to modulate the gut microbiome for enhanced immunity outcomes. For instance, the practice of providing newborn piglets with oral supplements of “engineered” bacterial cultures has been applied to outcompete those pathogenic bacteria. Additionally, optimization of feed and feed additives resulted in increased survival rates for weaning piglets. Ultimately, integrating the insights of the effects of nutrients on gut microbiota into swine nutrition and veterinary practices will contribute to a holistic approach that prioritizes both the nutritional needs and the well-being of pigs in a sustainable manner for pork production.

## 6. Conclusions

Practically, during the course of pork production, swine diets are modulated to obtain desirable production outcomes while also minimizing all other non-feed costs. Nutritional strategies to manipulate the intestinal microbial ecosystem are useful measures to optimize gut health and function. Current knowledge about the effects of dietary AA supplementation on the composition and metabolism of gut bacteria, as reviewed in this paper, should be considered a useful nutritional management strategy for swine production. In particular, providing pigs with nutrients that promote the growth of *Lactobacillus* and *Bifidobacterium* can lead to better gut health, gut functions, and growth performance, which may also be achieved through the dietary addition of NAC. One example for manipulating gut microbiota is dietary supplementation with BCAA, especially when the dietary protein is limited, since BCAA can alter the gut microbiota composition, which will consequently alleviate the negative impact of LP diets on pig performance.

Although our current knowledge on the effects of dietary AA on gut microbiota allows us to make predictions and test hypotheses regarding the expected outcomes on animal performance, the effects may be better if the AA strategy is considered in conjunction with some other nutritional measures, including the provision of probiotics, prebiotics, and/or postbiotics. A combinational use of AA with probiotics, prebiotics, and/or postbiotics should be investigated in future work for the provision of better feed additives and for their synergistic effects on gut health and growth performance.

Since different groups of commensal microorganisms contribute different metabolites to the overall metabolic pool of the host, microorganisms in the gut do not act individually but rather form consortia to produce the metabolites, even though the functional roles of individual microbial species need to be investigated at least at present. More work, therefore, is immediately needed to understand the composition and functions of the diverse microbiome in the gut, including further descriptions of the microbial communities present and further elucidation of the mechanisms arising from dietary AA manipulation that results in desirable outcomes in growth performance. Establishing those mechanistic cause-and-effect relationships should allow swine producers to reap the greatest return on their feed investment.

## Data Availability

Not applicable.

## Supplementary Materials

The supporting information can be downloaded at: https://www.mdpi.com/article/10.3390/ijms25021237/s1, references [153,192,193,194,195,196,197,198,199,200,201,202,203] are cited in Supplementary Materials.
