# Supplementary material for: Swine Gastrointestinal Microbiota and the Effects of Dietary Amino Acids on Its Composition and Metabolism
Source: Int J Mol Sci. 2024 Jan 19;25(2):1237. doi: 10.3390/ijms25021237 (PMC10816286; doi:10.3390/ijms25021237)
Supplement: Supplementary file 1 [file ijms-25-01237-s001.zip › Supplemental Table S1.pdf]

Supplemental Table S1. A taxonomical list of the named bacteria commonly presented in swine gastrointestinal tract as reported in the literature [1,2]

Phylum **Class** **Order** Family *Genus* *Species* (References)

Actinomycetota (Actinobacteriota)

Actinomycetes (Actinobacteria)

Actinomycetales

Actinomycetaceae

Actinomyces

bovis; hyovaginalis; suimastitidis; urogenitalis

(Wylensek et al., 2020; Holman et al., 2022; Tan et al., 2017)

Mobiluncus

curtisii; mulieris; porci

(Tan et al., 2017; Holman et al., 2022)

Trueperella

pyogenes

(Tan et al., 2017; Holman et al., 2022)

Bifidobacteriales

Bifidobacteriaceae

Bifidobacterium

animalis; asteroides; biavatii; boum; globosum; longum; kashiwanohense; merycicum; pseudolongum; saeculare; thermacidophilum; thermophilum; tsurumiense

(Leser et al., 2002; Dowd et al., 2008; Tan et al., 2017; Wylensek et al., 2020, Holman et al., 2022; Gaio et al., 2021)

Catenulisporales

Catenulisporaceae

Catenulispora

Acidiphila

(Yang et al., 2017)

Geodermatophilales

Geodermatophilaceae

Blastococcus

saxobsidens

(Yang et al., 2017)

Mycobacteriales (Corynebacteriales)

Corynebacteriaceae

Corynebacterium

ammoniagenes; diphtheriae; glucuronolyticum; kroppenstedtii; pseudotuberculosis; stationis; striatum; variabile; xerosis

(Dowd et al., 2008; Tan et al., 2017; Wang et al., 2019; Wylensek et al., 2020)

Mycobacteriaceae (Mycobactericidal)

Mycobacterium

tuberculosis

(Quan et al., 2020)

Nocardiaceae

Rhodococcus

coprophilus

(Tan et al., 2017)

Micrococcales

Micrococcaceae

Arthrobacter

sp.

(Wylensek et al., 2020)
Kocuria
sp.
(Dowd et al., 2008)
Micrococcus
luteus
(Wylensek et al., 2020)
Rothia
sp.
(Dowd et al., 2008)
Promicromonosporaceae
Aneriacter
sp.
(Luo et al., 2022)
Cellulosimicrobium
aquatile; cellulans
(Wang et al., 2019; Wylensek et al., 2020)
Xylanimonas
cellulosilytica
(Yang et al., 2017)

Micromonosporales
Micromonosporaceae
Actinoplanes
*friuliensis; liguriensis; missour*iensis
(Yang et al., 2017)

Nakamurellales
Nakamurellaceae

Nakamurella

multipartite

(Yang et al., 2017)

Propionibacteriales

Propionibacteriaceae

Propionibacterium

sp.

(Collado and Sanz, 2007)

Kitasatosporales

Streptomycetaceae

Streptomyces

xinghaiensis; koyangensis

(Tan et al., 2017; Wang et al., 2019)

Coriobacteriia

Coriobacteriales

Atopobiaceae

Atopobium

*deltae; parvu*lum

(Dowd et al., 2008; Wang et al., 2019; Wylensek et al., 2020)

Olsenella

porci; umbonate

(Dowd et al., 2008; Wylensek et al., 2020)

Parafannyhessea

umbonate

(Holman et al., 2022)

Paratractidigestivibacter

faecalis

(Holman et al., 2022)
Parolsenella
sp.
(Holman et al., 2022)
Tractidigestivibacter
sp.
(Holman et al., 2022)
Coriobacteriaceae
Collinsella
aerofaciens; bouchesdurhonensis; tanakaei
(Wylensek et al., 2020; Tan et al., 2017; Holman et al., 2022; Ortiz Sanjuán et al., 2022)
Enorma
massiliensis
(Tan et al., 2017; Holman et al., 2022)

Eggerthellales

Eggerthellaceae
Denitrobacterium
detoxificans
(Tan et al., 2017; Wylensek et al., 2020)
Eggerthella
l*enta*
(Tan et al., 2017; Wylensek et al., 2020)

Thermoleophilia

Solirubrobacterales

Conexibacteraceae
Conexibacter
woesei

(Yang et al., 2017)

Bacillota (Firmicutes)

Bacilli

Bacillales

Bacillaceae

Bacillus

altitudinis; sporothermodurans; velezensis

(Leser et al., 2002; Dowd et al., 2008; Quan et al., 2020; Wylensek et al., 2020)

Paucisalibacillus

sp.

(Yin et al., 2018)

Psychrobacillus

sp.

(Wylensek et al., 2020)

Bacillales Family XI. Incertae Sedis[3]

Gemella

sp.

(Dowd et al., 2008)

Paenibacillaceae

Aneurinibacillus

aneurinilyticus

(Wylensek et al., 2020)

Brevibacillus

sp.

(Goodarzi et al., 2022)

Planococcaceae

Kurthia

huakuii

(Yang et al., 2017)

Solibacillus

silvestris

(Wang et al., 2019)

Staphylococcaceae

Staphylococcus

aureus; capitis; cohnii; epidermidis; haemolyticus; hominis; phage; schleiferi

(Robinson et al. 1981; Dowd et al., 2008; Quan et al., 2020; Tan et al., 2017; Wylensek et al., 2020)

Lactobacillales

Aerococcaceae

Abiotrophia

sp.

(Dowd et al., 2008)

Aerococcus

suis; urinaeequi

(Dowd et al., 2008; Holman et al., 2022)

Globicatella

sp.

(Dowd et al., 2008)

Carnobacteriaceae

Carnobacterium

sp.

(Dowd et al., 2008; Tan et al., 2017)

Granulicatella

sp.

(Dowd et al., 2008; Tan et al., 2017)

Enterococcaceae

Enterococcus

cecorum; faecalis; faecium; gallinarum; hirae

(Dowd et al., 2008; Holman et al., 2022; Wylensek et al., 2020)

Tetragenococcus

sp.

(Dowd et al., 2008)

Vagococcus

teuberi

(Wang et al., 2019)

Lactobacillaceae

Lactobacillus

acidophilus; agilis; amylophilus; amylovorus; animalis; backii; crispatus; delbrueckii; equicursoris; fermentum; gallinarum; helveticus; ingluviei; johnsonii; kitasatonis; mucosae; paralimentarius; pasteurii; plantarum; porci; reuteri; ruminis; salivarius

(Robinson et al. 1981, 2005; Pryde et al., 1999; Leser et al., 2002; Li et al., 2003; Hill et al., 2005; Dowd et al., 2008; Dai et al. 2010; Li and Young, 2013; Kelly et al., 2017; Quan et al., 2018; Tan et al., 2017; Wylensek et al., 2020; Gaio et al., 2021; Liu et al., 2021; Holman et al., 2022)

Ligilactobacillus

agilis; animalis; salivarius; ruminis

(Holman et al., 2022)

Limosilactobacillus

coleohominis; mucosae; reuteri; vaginalis

(Holman et al., 2022)

Oenococcus

oeni

(Tan et al., 2017)

Pediococcus

damnosus

(Hill et al., 2005; Tan et al., 2017)

Weissella

sp.

(Dowd et al., 2008)

Streptococcaceae

Lactococcus

garvieae; lactis

(Dowd et al., 2008; Li and Young, 2013; Ortiz Sanjuán et al., 2022)

Streptococcus

alactolyticus; anginosus; constellatus; criceti; equinus; faecium; gallolyticus; hyointestinalis; hyovaginalis; infantarius; intermedius; lutetiensis; macedonicus; orisratti; pasteurianus; pneumoniae; pyogenes; suis; thermophilus; vestibularis

(Robinson et al. 1981, 2005; Dowd et al., 2008; Dai et al., 2010; Li and Young, 2013; Quan et al., 2018; Tan et al., 2017; Wang et al., 2019; Wylensek et al., 2020; Liu et al., 2021; Holman et al., 2022)

Bacillota sensu stricto incertae sedis[3]

Order?

Family?

Negativibacillus

massiliensis

(Holman et al., 2022)

Clostridia

Eubacteriales

Anaerovoracaceae

Genus?

sp.

(Reyer, Henry, et al., 2021)

Cellulosilyticaceae

Cellulosilyticum

sp.

(Gao et al., 2023)

Christensenellaceae

Catabacter

hongkongensis

(Tan et al., 2017)

Christensenella

minuta

(Tan et al., 2017)

Clostridiaceae

Butyricicoccus

porcorum; pullicaecorum

(Tan et al., 2017; Holman et al., 2022)

Clostridium

asparagiforme; beijerinckii; bolteae; botulinum; butyricum; cadaveris; celerecrescens; cellulosi; citroniae; clostridioforme; cochlearium; difficile; indolis; innocuum; methylpentosum; perfringens; porci; saccharolyticum; scindens; sphenoides; sporosphaeroides; symbiosum

(Pryde et al., 1999; Leser et al., 2002; Dowd et al., 2008; Isaacson and Kim, 2012; Quan et al., 2018, 2020; Wylensek et al., 2020; Holman et al., 2022)

Hungatella

hathewayi

(Tan et al., 2017; Holman et al., 2022)

Natronincola

sp.
(Dowd et al., 2008)
Oxobacter
sp.
(Leser et al., 2002)
Paraclostridium
sp.
(Wylensek et al., 2020)
Sarcina
maxima
(Leser et al., 2002; Dowd et al., 2008)
Desulfitobacteriaceae
Desulfitobacterium
sp.
(Leser et al., 2002)
Syntrophobotulus
sp.
(Leser et al., 2002)
Desulfitomaculaceae
Desulfitomaculum
sp.
(Leser et al., 2002)
Pelotomaculum
sp.
(Tan et al., 2017)
Eubacteriaceae
Eubacterium

brachy; callanderi; coprostanoligenes; hallii; innocuum; limosum; plexicaudatum; pyruvativorans; ramulus; rectale; xylanophilum

(Robinson et al. 1981; Pryde et al., 1999; Leser et al., 2002; Dowd et al., 2008; Tan et al., 2017; Liu et al., 2021; Ortiz Sanjuán et al., 2022; Holman et al., 2022)

Pseudoramibacter

sp.

(Leser et al., 2002)

Eubacteriales incertae sedis[3]

Gemmiger

formicilis

(Liu et al., 2021; Holman et al., 2022)

Intestinimonas

butyriciproducens; massiliensis

(Tan et al., 2017; Holman et al., 2022; Ortiz Sanjuán et al., 2022)

Eubacteriales Family XII. Incertae Sedis

Acidaminobacter

sp.

(Leser et al., 2002)

Eubacteriales Family XIII. Incertae Sedis

Anaerovorax

sp.

(Gao et al., 2023)

Emergencia

sp.

(Holman et al., 2022)

Mogibacterium

sp.

(Wylensek et al., 2020)

Lachnospiraceae

Acetitomaculum

sp.

(Leser et al., 2002)

Agathobacter

sp.

(Holman et al., 2022)

Anaerobutyricum

soehngenii

(Wylensek et al., 2020; Holman et al., 2022)

Anaerostipes

caccae; hadrus

(Tan et al., 2017; Holman et al., 2022; Ortiz Sanjuán et al., 2022)

Anaerotignum

sp.

(Holman et al., 2022)

Bariatricus

sp.

(Holman et al., 2022)

Bilifractor

sp.

(Holman et al., 2022)

Blautia

hansenii; hydrogenotrophica; massiliensis; obeum; producta; schinkii; wexlerae

(Tan et al., 2017; Holman et al., 2022; Ortiz Sanjuán et al., 2022)

Butyribacter

sp.

(Holman et al., 2022)

Butyrivibrio

crossotus; fibrisolvens; hungatei; proteoclasticus

(Robinson et al. 1981; Leser et al., 2002; Holman et al., 2022; Ortiz Sanjuán et al., 2022)

Coprococcus

catus; comes; eutactus; nexilis

(Leser et al., 2002; Tan et al., 2017; Liu et al., 2021; Holman et al., 2022)

Cuneatibacter

sp.

(Holman et al., 2022)

Dorea

formicigenerans; longicatena

(Dowd et al., 2008; Tan et al., 2017; Holman et al., 2022)

Eisenbergiella

sp.

(Holman et al., 2022)

Enterocloster

aldenensis; clostridioformis

(Holman et al., 2022)

Epulopiscium

sp.

(Leser et al., 2002)

Faecalicatena

contorta; faecis; glycyrrhizinilyticum; gnavus; lactaris; orotica; torques

(Gaio et al., 2021)

Faecalimonas

umbilicate

(Holman et al., 2022)

Fusicatenibacter

saccharivorans
(Holman et al., 2022)
Johnsonella
sp.
(Leser et al., 2002)
Kineothrix
sp.
(Holman et al., 2022)
Lachnoanaerobaculum
saburreum
(Tan et al., 2017)
Lachnobacterium
sp.
(Dowd et al., 2008)
Lachnoclostridium
mordavella; phocaeense; phytofermentans
(Tan et al., 2017; Wylensek et al., 2020; Liu et al., 2021; Ortiz Sanjuán et al., 2022)
Lachnospira
sp.
(Dowd et al., 2008; Holman et al., 2022)
Marvinbryantia
sp.
(Holman et al., 2022)
Mediterraneibacter
faecis
(Holman et al., 2022)
Muricomes
sp.

(Holman et al., 2022)

Oribacterium

sp.

(Holman et al., 2022)

Parasporobacterium

sp.

(Dowd et al., 2008)

Pseudobutyrivibrio

ruminis

(Leser et al., 2002)

Roseburia

faecis; hominis; intestinalis; inulinivorans

(Dowd et al., 2008; Kelly et al., 2017; Tan et al., 2017; Liu et al., 2021; Holman et al., 2022; Ortiz Sanjuán et al., 2022)

[Ruminococcus]

gnavus; lactaris; torques

(Holman et al., 2022)

Schaedlerella

sp.

(Holman et al., 2022)

Sporofaciens

sp.

(Holman et al., 2022)

Stomatobaculum

longum

(Tan et al., 2017)

Velocimicrobium

porci

(Wylensek et al., 2020)
Weimeria
sp.
(Holman et al., 2022)
Oscillospiraceae
Acetivibrio
ethanolgignens
(Dowd et al., 2008; Tan et al., 2017)
Agathobaculum
butyriciproducens
(Tan et al., 2017; Holman et al., 2022)
Anaerofilum
sp.
(Leser et al., 2002)
Anaerotruncus
sp.
(Tan et al., 2017)
Angelakisella
sp.
(Gaio et al., 2021)
Dysosmobacter
sp.
(Gaio et al., 2021; Holman et al., 2022)
Flavonifractor
plautii
(Holman et al., 2022; Ortiz Sanjuán et al., 2022)
Faecalibacterium
prausnitzii

(Dowd et al., 2008; Kelly et al., 2017; Tan et al., 2017; Liu et al., 2021; Holman et al., 2022)

Fournierella

excrementavium

(Holman et al., 2022)

Lawsonibacter

sp.

(Holman et al., 2022)

Monoglobus

sp.

(Holman et al., 2022)

Oscillibacter

ruminantium; valericigenes

(Tan et al., 2017)

Oscillospira

sp.

(Dowd et al., 2008)

Pseudoflavonifractor

capillosus

(Holman et al., 2022)

Pygmaiobacter

sp.

(Holman et al., 2022)

Ruminococcus

bromii; callidus; champanellensis; flavefaciens; gauvreauii

(Pryde et al., 1999; Leser et al., 2002; Dowd et al., 2008; Quan et al., 2020; Tan et al., 2017; Liu et al., 2021; Holman et al., 2022)

Ruthenibacterium

lactatiformans
(Hu et al., 2022)
Sporobacter
sp.
(Leser et al., 2002)
Subdoligranulum
variabile
(Liu et al., 2021; Holman et al., 2022)
Peptococcaceae
Peptococcus
sp.
(Leser et al., 2002)
Peptostreptococcaceae
Clostridioides
difficile
(Quan et al., 2020)
Intestinibacter
sp.
(Holman et al., 2022)
Peptostreptococcus
anaerobius
(Robinson et al. 1981; Pryde et al., 1999; Dowd et al., 2008; Tan et al., 2017)
Romboutsia
sp.
(Quan et al., 2020; Wang et al., 2019; Holman et al., 2022)
Terrisporobacter
ilealis; timonensis
(Liu et al., 2021; Holman et al., 2022)

Erysipelotrichia

Erysipelotrichales

Coprobacillaceae

Catenibacterium

mitsuokai

(Dowd et al., 2008; Holman et al., 2022)

Erysipelatoclostridium

ramosum

(Holman et al., 2022)

Sharpea

sp.

(Wylensek et al., 2020)

Erysipelotrichaceae

Absicoccus

porci

(Holman et al., 2022)

Allobaculum

stercoricanis

(Tan et al., 2017)

Bulleidia

sp.

(Dowd et al., 2008; Holman et al., 2022)

Catenisphaera

sp.

(Gao et al., 2023)

Faecalicoccus

pleomorphus

(Holman et al., 2022)
Faecalibaculum
rodentium
(Ortiz Sanjuán et al., 2022)
Faecalitalea
cylindroides; pleomorphus
(Tan et al., 2017; Wylensek et al., 2020)
Floccifex
porci
(Holman et al., 2022)
Holdemanella
biformis; porci
(Gaio et al., 2021; Holman et al., 2022)
Holdemania
sp.
(Leser et al., 2002)
Solobacterium
moorei
(Gaio et al., 2021)
Turicibacteraceae
Turicibacter
sanguinis
(Dowd et al., 2008; Wang et al., 2019; Quan et al., 2020)

Negativicutes

Acidaminococcales

Acidaminococcaceae
Acidaminococcus

fermentans

(Leser et al., 2002; Dowd et al., 2008; Dai et al. 2010; Tan et al., 2017; Wylensek et al., 2020; Ortiz Sanjuán et al., 2022)

Phascolarctobacterium

succinatutens

(Leser et al., 2002; Tan et al., 2017; Gaio et al., 2021; Holman et al., 2022)

Succiniclasticum

sp.

(Leser et al., 2002; Holman et al., 2022)

Selenomonadales

Selenomonadaceae

Mitsuokella

jalaludinii

(Holman et al., 2022)

Pectinatus

sp.

(Leser et al., 2002)

Selenomonas

bovis; ruminantium

(Robinson et al., 1981; Pryde et al., 1999; Isaacson and Kim, 2012; Tan et al., 2017; Liu et al., 2021; Holman et al., 2022)

Sporomusaceae

Sporomusa

sp.

(Leser et al., 2002)

Veillonellales

Veillonellaceae

Caecibacter

massiliensis
(Wylensek et al., 2020; Holman et al., 2022)
Dialister
succinatiphilus
(Leser et al., 2002)
Megasphaera
elsdenii; hexanoica
(Wylensek et al., 2020; Holman et al., 2022)
Veillonella
atypica; caviae; seminalis
(Li et al., 2003; Dowd et al., 2008; Tan et al., 2017)

Tissierellia

Tissierellales

Peptoniphilaceae
Anaerovibrio
lipolytica; slackiae
(Dowd et al., 2008; Dai et al. 2010; He et al., 2016; Wylensek et al., 2020; Holman et al., 2022)
Anaerococcus
sp.
(Dowd et al., 2008)
Finegoldia
magna
(Dowd et al., 2008)
Parvimonas
sp.
(Holman et al., 2022)

Peptoniphilus

sp.

(Dowd et al., 2008)

Sporanaerobacteraceae

Anaerosalibacter

bizertensis

(Wylensek et al., 2020)

Tissierellia incertae sedis[3]

Ezakiella

sp.

(Holman et al., 2022)

Bacteroidota (Bacteroidetes)

Bacteroidia

Bacteroidales

Bacteroidaceae

Bacteroides

*caecimuris; dorei; eggerthii; fragilis; helcogenes; heparinolyticus; ovatus; pyogenes; salanitronis; stercoris; thetaiotaomicron; uniformis; vulgatus; xylanisol*vens

(Robinson et al., 1981; Pryde et al., 1999; Leser et al., 2002; Dowd et al., 2008; Wylensek et al., 2020; Holman et al., 2022; Ortiz Sanjuán et al., 2022)

Phocaeicola

plebeius; vulgatus

(Holman et al., 2022)

Barnesiellaceae

Barnesiella

intestinihominis; viscericola

(Yang et al., 2017)

Dysgonomonadaceae
 Butyricimonas
 virosa
 (Holman et al., 2022)
 Fermentimonas
 caenicola
 (Tan et al., 2017)
 Odoribacter
 sp.
 (Holman et al., 2022)
 Proteiniphilum
 saccharofermentans
 (Yang et al., 2017)
Muribaculaceae
 Duncaniella
 sp.
 (Kim et al., 2023)
Paludibacteraceae
 Paludibacter
 sp.
 (Kim et al., 2023)
Porphyromonadaceae
 Porphyromonas
 sp.
 (Leser et al., 2002; Tan et al., 2017; Holman et al., 2022)
Prevotellaceae
 Alloprevotella
 sp.

(Quan et al., 2018)

Hallella

seregens

(Tan et al., 2017)

Paraprevotella

sp.

(Quan et al., 2020)

Prevotella

copri; dentalis; enoeca; fusca; intermedia; jejuni; melaninogenica; nigrescens; oralis; ruminicola

(Leser et al., 2002; Isaacson and Kim, 2012; Kelly et al., 2017; Zhang et al., 2018; Tan et al., 2017; Quan et al., 2020; Wylensek et al., 2020; Ortiz Sanjuán et al., 2022)

Rikenellaceae

Alistipes

senegalensis; shahii

(Quan et al., 2020; Liu et al., 2021; Holman et al., 2022)

Rikenella

sp.

(Leser et al., 2002)

Tannerellaceae

Parabacteroides

distasonis; faecavium; merdae

(Quan et al., 2020; Wylensek et al., 2020; Holman et al., 2022)

Tannerella

sp.

(Tan et al., 2017)

Marinilabiliales

Marinifilaceae

Marinifilum

sp.

(Bai et al., 2021)

Cytophagia

Cytophagales

Cytophagaceae

Cytophaga

sp.

(Leser et al., 2002)

Flexibacteraceae

Flexibacter

sp.

(Leser et al., 2002)

Flavobacteriia

Flavobacteriales

Weeksellaceae

Bergeyella

sp.

(Dowd et al., 2008)

Chryseobacterium

gallinarum

(Dowd et al., 2008; Ortiz Sanjuán et al., 2022)

Campylobacterota

Epsilonproteobacteria

Campylobacterales

Campylobacteraceae

Campylobacter

coli; hominis; lanienae; jejuni

(Dowd et al., 2008; Kelly et al., 2017; Holman et al., 2022)

Helicobacteraceae

Helicobacter

bilis; mustelae; trogontum

(Dowd et al., 2008; Kelly et al., 2017; Tan et al., 2017; Holman et al., 2022)

Chlamydiota

Chlamydiia

Chlamydiales

Chlamydiaceae

Chlamydia

suis; trachomatis

(Quan et al., 2020; Tan et al., 2017; Liu et al., 2021; Holman et al., 2022)

Cyanobacteriota (Cyanobacteria or Cyanophyta)

Cyanophyceae

Oscillatoriales

Microcoleaceae

Microcoleus

sp.

(Yang et al., 2017)

Deferribacterota

Deferribacteres

Deferribacterales

Flexistipitaceae

Flexistipes

sp.

(Leser et al., 2002)

Mucispirillaceae

Mucispirillum

faecigallinarum

(Holman et al., 2022)

Elusimicrobiota (Termite Group 1)

Elusimicrobia

Elusimicrobiales

Elusimicrobiaceae

Elusimicrobium ?

sp.

(Holman et al., 2022)

Fibrobacterota

Fibrobacteria

Fibrobacterales

Fibrobacteraceae

Fibrobacter

intestinalis; succinogenes

(Dowd et al., 2008; Wylensek et al., 2020; Ortiz Sanjuán et al., 2022)

Hallerella

sp.

(Holman et al., 2022)

Fusobacteriota

Fusobacteriia

Fusobacteriales

Fusobacteriaceae

Fusobacterium

mortiferum; necrophorum; perfoetens; varium

(Robinson et al. 1981; Gaskins 2001; Leser et al., 2002; Dowd et al., 2008; Wylensek et al., 2020)

Leptotrichiaceae

Leptotrichia

buccalis

(Robinson et al. 1981; Dowd et al., 2008)

Gemmatimonadota

Gemmatimonadetes

Gemmatimonadales

Gemmatimonadaceae

Gemmatimonas

aurantiaca

(Yang et al., 2017)

Lentisphaerota (Lentisphaerae)

Lentisphaeria

Victivallales

Victivallaceae

Victivallis
vadensis
(Holman et al., 2022)

Mycoplasmatota (Tenericutes)

Mollicutes

Acholeplasmatales

Acholeplasmataceae
Acholeplasma
sp.
(Leser et al., 2002)

Anaeroplasmatales

Anaeroplasmataceae
Anaeroplasma
sp.
(Holman et al., 2022)
Asteroleplasma
sp.
(Dowd et al., 2008; Holman et al., 2022)

Entomoplasmatales

Spiroplasmataceae
Spiroplasma
sp.
(Dowd et al., 2008)

Mycoplasmatales

Mycoplasmataceae
Mycoplasma

sp.
(Leser et al., 2002; Tan et al., 2017; Liu et al., 2021)

Planctomycetota (Planctomycetes)

Planctomycetia

Planctomycetales

Planctomycetaceae

Planctomyces

sp.

(Leser et al., 2002)

Pseudomonadota (Proteobacteria)

Alphaproteobacteria

Hyphomicrobiales

Beijerinckiaceae

Beijerinckia

indica

(Yang et al., 2017)

Methylocapsa

acidiphila

(Yang et al., 2017)

Nitrobacteraceae

Nitrobacter

hamburgensis; winogradskyi

(Yang et al., 2017)

Rhodopseudomonas

sp.

(Dowd et al., 2008)
Parvibaculaceae
Parvibaculum
lavamentivorans
(Yang et al., 2017)
Rhizobiaceae
Allorhizobium
sp.
(Yang et al., 2017)
Ensifer
sp.
(Dowd et al., 2008)
Neorhizobium
galegae
(Yang et al., 2017)
Pararhizobium
sp.
(Yang et al., 2017)
Rhizobium
oryzae
(Yang et al., 2017)
Xanthobacteraceae
Starkeya
novella
(Yang et al., 2017)

Rhodobacterales
Paracoccaceae
Rhodobacter

sp.
(Dowd et al., 2008)
Rhodovulum
sp.
(Dowd et al., 2008)

Rhodospirillales

Azospirillaceae
Azospirillum
brasilense; lipoferum
(Yang et al., 2017)

Sphingomonadales

Sphingomonadaceae
Sphingobium
sp.
(Tan et al., 2017)
Sphingomonas
sp.
(Li et al., 2019)

Betaproteobacteria

Burkholderiales

Comamonadaceae
Alicycliphilus
denitrificans
(Yang et al., 2017)
Aquabacterium
sp.
(Yin et al., 2018)

Comamonas

kerstersii; testosteroni

(Yang et al., 2017; Wang et al., 2019)

Sutterellaceae

Mesosutterella

sp.

(Holman et al., 2022)

Sutterella

parvirubra; wadsworthensis

(Holman et al., 2022)

Neisseriales

Neisseriaceae

Neisseria

perflava

(Holman et al., 2022)

Oxalobacteraceae

Massilia

sp.

(Li et al., 2019)

Oxalobacter

formigenes

(Tan et al., 2017)

Rhodocyclales

Rhodocyclaceae

Genus?

sp.

(Reddy et al., 2021)

Zoogloeaceae

Thauera

selenatis

(Tan et al., 2017)

Gammaproteobacteria

Aeromonadales

Succinivibrionaceae

Anaerobiospirillum

succiniciproducens

(Holman et al., 2022)

Succinivibrio

dextrinosolvens

(Dai et al., 2010)

Alteromonadales

Idiomarinaceae

Idiomarina

sp.

(Yang et al., 2017)?

Enterobacterales

Enterobacteriaceae

Citrobacter

rodentium

(Ortiz Sanjuán et al., 2022)

Enterobacter

asburiae

(Isaacson and Kim, 2012)

Escherichia

coli; fergusonii; flexneri

(Leser et al., 2002; Dai et al., 2010; Kelly et al., 2017; Wylensek et al., 2020; Tan et al., 2017; Wang et al., 2019; Gaio et al., 2021; Holman et al., 2022)

Klebsiella

pneumoniae

(Dai et al., 2010; Wang et al., 2019)

Pluralibacter

gergoviae

(Tan et al., 2017)

Salmonella

enterica

(Wang et al., 2019; Dowd et al., 2008)

Shigella

boydii; sonnei

(Li et al., 2003)

Hafniaceae

Hafnia

alvei

(Li and Young, 2013)

Morganellaceae

Photorhabdus

luminescens

(Tan et al., 2017)

Morganella

morganii

(Li and Young, 2013)

Vibrionaceae

Vibrio

sp.
(Mukhopadhya et al., 2012)
Yersiniaceae
Serratia
marcescens
(Tan et al., 2017)

Moraxellales

Moraxellaceae
Acinetobacter
sp.
(Dowd et al., 2008; Tan et al., 2017)

Pasteurellales

Pasteurellaceae
Actinobacillus
porcinus
(Holman et al., 2022)
Haemophilus
parainfluenzae; pittmaniae
(Tan et al., 2017)
Pasteurella
bettyae
(Tan et al., 2017)

Pseudomonadales

Pseudomonadaceae
Pseudomonas
putida
(Leser et al., 2002; Dai et al., 2010; Tan et al., 2017)

Xanthomonadales

Xanthomonadaceae

Stenotrophomonas

sp.

(Dowd et al., 2008)

Spirochaetota

Spirochaetia

Sphaerochaetales

Sphaerochaetaceae

Bullifex

porci

(Holman et al., 2022)

Spirochaetales

Spirochaetaceae

Spirochaeta

sp.

(Dowd et al., 2008; Tan et al., 2017; Wylensek et al., 2020)

Treponemataceae

Treponema

berlinense; brennaborense; bryantii; porcinum; succinifaciens; vincentii

(Leser et al., 2002; Nordhoff et al., 2005; Quan et al., 2020; Wylensek et al., 2020; Liu et al., 2021; Holman et al., 2022)

Synergistota

Synergistia

Synergistales

Acetomicrobiaceae

Acetomicrobium

Anaerobaculum

(Leser et al., 2002)

Dethiosulfovibrionaceae

Pyramidobacter

sp.

(Wylensek et al., 2020; Holman et al., 2022)

Synergistaceae

Cloacibacillus

porcorum

(Wang et al., 2019; Wylensek et al., 2020; Holman et al., 2022)

Thermodesulfobacteriota

Desulfovibrionia

Desulfovibrionales

Desulfovibrionaceae

Bilophila

wadsworthia

(Gaio et al., 2021; Holman et al., 2022)

Desulfovibrio

piger; porci

(Wylensek et al., 2020)

Lawsonia

intracellularis

(Quan et al., 2020)

Desulfuromonadia

Geobacterales

Geobacteraceae

Geobacter

soli

(Tan et al., 2017)

Verrucomicrobiota

Verrucomicrobiota (Verrucomicrobia)

Verrucomicrobiales

Akkermansiaceae

Akkermansia

muciniphila

(Holman et al., 2022)

---

[1] It is better to read this table in "Web Layout" view and with "Navigation Pane" open for a quick navigation. For each taxonomical level (Phylum, Class, Order, Family, Genus, or Species), the names are listed in the alphabetic order. Each of the levels (except for the Species level) can be collapsed or expanded by hovering your computer-mouse pointer to the immediate left of the level name until a small black triangle showing up, and then clicking on this small black triangle.

[2] The nomenclature and classification of this table followed the NCBI Taxonomy housed in National Library of Medicine (https://www.ncbi.nlm.nih.gov/taxonomy). The words in parentheses after those taxonomic names are the corresponding names previously used, and in fact many of them are currently still in use in the literature.

[3] This phrase, incertae sedis, indicates that the name associated with it is an uncertain taxonomic rank or name.
